# The 2021 *Nucleic Acids Research* database issue and the online molecular biology database collection

**DOI:** 10.1093/nar/gkaa1216

**Published:** 2020-12-23

**Authors:** Daniel J Rigden, Xosé M Fernández

**Affiliations:** Institute of Systems, Molecular and Integrative Biology, University of Liverpool, Crown Street, Liverpool L69 7ZB, UK; Institut Curie, 25 rue d’Ulm, 75005 Paris, France

## Abstract

The 2021 *Nucleic Acids Research* database Issue contains 189 papers spanning a wide range of biological fields and investigation. It includes 89 papers reporting on new databases and 90 covering recent changes to resources previously published in the Issue. A further ten are updates on databases most recently published elsewhere. Seven new databases focus on COVID-19 and SARS-CoV-2 and many others offer resources for studying the virus. Major returning nucleic acid databases include NONCODE, Rfam and RNAcentral. Protein family and domain databases include COG, Pfam, SMART and Panther. Protein structures are covered by RCSB PDB and dispersed proteins by PED and MobiDB. In metabolism and signalling, STRING, KEGG and WikiPathways are featured, along with returning KLIFS and new DKK and KinaseMD, all focused on kinases. IMG/M and IMG/VR update in the microbial and viral genome resources section, while human and model organism genomics resources include Flybase, Ensembl and UCSC Genome Browser. Cancer studies are covered by updates from canSAR and PINA, as well as newcomers CNCdatabase and Oncovar for cancer drivers. Plant comparative genomics is catered for by updates from Gramene and GreenPhylDB. The entire Database Issue is freely available online on the *Nucleic Acids Research* website (https://academic.oup.com/nar). The NAR online Molecular Biology Database Collection has been substantially updated, revisiting nearly 1000 entries, adding 90 new resources and eliminating 86 obsolete databases, bringing the current total to 1641 databases. It is available at https://www.oxfordjournals.org/nar/database/c/.

## NEW AND UPDATED DATABASES

The 28th annual *Nucleic Acids Research* Database Issue contains 189 papers spanning, as usual, a wide range of biology. Unsurprisingly, COVID-19 casts a long shadow over the Issue. Seven new databases specifically address the pandemic and the SARS-CoV-2 virus responsible (Table 1) but new and returning databases in all areas have rushed to support research into the viral pandemic: the reader will find reference to it throughout the Issue, sometimes in quite unexpected places. The Issue contains a further 82 papers (Table 2) on new databases as well as 90 update papers on databases previously published in NAR. To complete the Issue, resources previously published elsewhere update in a further 10 papers (Table 3).

**Table 1.** Descriptions of new databases related to COVID-19 in the 2021 *NAR* database Issue

Database name	URL	Short description
CoV3D	https://cov3d.ibbr.umd.edu/	Experimental coronavirus protein structures
COVID19 Drug Repository	http://covid19.md.biu.ac.il/	Curated papers on COVID-19 drugs and drug repurposing
DockCoV2	https://covirus.cc/drugs/	In silico drug docking against SARS-CoV2 targets
GESS	https://wan-bioinfo.shinyapps.io/GESS/	Global Evaluation of SARS-CoV2 Sequences
LitCovid	https://www.ncbi.nlm.nih.gov/research/coronavirus/	Curated COVID-19 literature
PAGER-COV	http://discovery.informatics.uab.edu/PAGER-COV/	Pathways and gene lists related to COVID-19
ViruSurf	http://gmql.eu/virusurf/	Portal to SARS-CoV2 sequences and variants

**Table 2.** Descriptions of new databases in the 2021 *NAR* database Issue not specifically related to COVID-19

Database name	URL	Short description
Aging Atlas	https://bigd.big.ac.cn/aging	Aging related omics data
Animal-APAdb	http://gong_lab.hzau.edu.cn/Animal-APAdb/	Alternative polyadenylation in animals
AcrDB	http://bcb.unl.edu/AcrDB/	Anti-CRISPR operons
AcrHub	http://pacrispr.erc.monash.edu/AcrHub	Anti-CRISPR proteins
ATACdb	http://www.licpathway.net/ATACdb	Human Assay-for-Transposase-Accessible Chromatin data
AtMAD	http://www.megabionet.org/atmad	Arabidopsis thaliana Multi-omics Association Database
BastionHub	http://bastionhub.erc.monash.edu/	Substrates of Gram-negative secretion systems
BiG-FAM	https://bigfam.bioinformatics.nl	Biosynthetic gene cluster families
CancerImmunityQTL	http://www.cancerimmunityqtl-hust.com/	ImmunQTLs across multiple cancer types
Chewie-NS	https://chewbbaca.online/	Gene-by-gene schemas for microbial strain identification
CMNPD	https://www.cmnpd.org/	Comprehensive Marine Natural Product Database
CNCDatabase	https://cncdatabase.med.cornell.edu/	Cancer drivers at non-coding regions
cncRNAdb	http://www.rna-society.org/cncrnadb/	Coding and non-coding RNA
ConjuPepDB	http://conjupepdb.ttk.hu/	Drug-peptide conjugates
CovInDB	http://cadd.zju.edu.cn/cidb/	Covalent Inhibitor DataBase
crisprSQL	http://www.crisprsql.com/	CRISPR/Cas9 Off-Target Cleavage Assays
CRISPR-view	http://crispviewdatabase.weililab.org/	Functional genetic screens
CSEA-DB	https://bioinfo.uth.edu/CSEADB/	Cell type specificity of genetic traits
CSVS	http://csvs.babelomics.org/	Spanish genomes and exomes
Cyanorak	http://www.sb-roscoff.fr/cyanorak	Comparative genomics of cyanobacteria
Datanator	https://datanator.info/	Molecular data for modeling biochemical networks
dbCAN-PUL	http://bcb.unl.edu/dbCAN_PUL/home	Experimentally characterized CAZyme gene clusters
dbGuide	https://sgrnascorer.cancer.gov/dbguide	Manually curated and functionally validated guide RNAs
DescribePROT	http://biomine.cs.vcu.edu/servers/DESCRIBEPROT/	Residue level prediction of structure and function across proteomes
DKK	https://darkkinome.org/	Dark Kinome Knowledgebase
DIGGER	https://exbio.wzw.tum.de/digger	Alternative splicing and protein-protein interactions
DNAmoreDB	http://www.genesilico.pl/DNAmoreDB	DNAzymes, i.e. DNA molecules with catalytic activity
DrugSpaceX	https://drugspacex.simm.ac.cn/	100 million compounds for virtual screening
DualSeqDB	http://www.tartaglialab.com/dualseq/	Dual RNA-seq host-pathogen sequencing
FireProtDB	https://loschmidt.chemi.muni.cz/fireprotdb	Protein stability data
gcType	http://gctype.wdcm.org/	WDCM 10K sequencing projects
GIMICA	https://idrblab.org/gimica/	Host Genetic and Immune Factors Shaping Human Microbiota
GlycoPOST	https://glycopost.glycosmos.org/	Raw Mass Spectrometry glycomics data
GRNdb	http://www.grndb.com/	TF-target relationships inferred from single cell and bulk RNA-seq datasets
GSRS	https://gsrs.ncats.nih.gov/app/substances	Global Substance Registration System
HeRA	https://hanlab.uth.edu/HeRA/	Human enhancer RNA Atlas
HERB	http://herb.ac.cn/	High-throughput Experiment- and Reference-guided dataBase of TCM
Housekeeping Transcript Atlas	http://www.housekeeping.unicamp.br/	Human and mouse housekeeping genes
HumanMetagenomeDB	https://webapp.ufz.de/hmgdb/	Curated and standardized metadata for human metagenomes
iCSDB	http://www.kobic.re.kr/icsdb	integrated CRISPR Screens DataBase
IDDB	http://mdl.shsmu.edu.cn/IDDB	Infertility Disease DataBase
iModulonDB	https://imodulondb.org/	‘iModulons’, groups of independently-modulated genes
IndiGenomes	http://clingen.igib.res.in/indigen/	Genetic variation in 1000 Indian individuals
INTEDE	https://idrblab.org/intede/	Interactome of Drug-Metabolizing Enzymes
KinaseMD	https://bioinfo.uth.edu/kmd/	Kinase Mutations and Drug responses
LnCeCell	http://bio-bigdata.hrbmu.edu.cn/LnCeCell/	lncRNA-associated ceRNA networks
LncExpDB	https://bigd.big.ac.cn/lncexpdb	Human lncRNA expression database
LncSEA	http://bio.liclab.net/LncSEA/	Reference human lncRNA sets
m6A-Atlas	http://www.xjtlu.edu.cn/biologicalsciences/atlas	The N6-methyladenosine (m6A) epitranscriptome including base-resolution data
markerDB	http://www.markerdb.ca/	Biomarkers: chemical, protein, chromosomal and genetic
MASI	http://www.aiddlab.com/MASI/	Microbiota – Active Substance Interactions database
MeDAS	https://das.chenlulab.com	Alternative splicing during development in 20 species
MemMoRF	http://memmorf.hegelab.org	Membrane-binding Molecular Recognition Features
mMGE	http://mgedb.comp-sysbio.org/	Human metagenomic extrachromosomal mobile genetic elements
MolluscDB	http://mgb.biocloud.net/home	Comparative genomics of molluscs
Nucleome Data Bank	https://ndb.rice.edu/	3D genome structures and simulations
OncoVar	https://oncovar.org/	Driver mutations, genes and pathways in cancer
Open Targets Genetics	https://genetics.opentargets.org/	Drug targets prioritised from genetic data
PCAT	http://pedtranscriptome.org	Pediatric cancer transcriptome explorer
Peryton	https://dianalab.e-ce.uth.gr/peryton	Microbe–disease associations
PheLiGe	https://phelige.com/	Genotype–phenotype associations
PhycoCosm	http://phycocosm.jgi.doe.gov/	Comparative genomics of algae
PK-DB	https://pk-db.com/	Pharmacokinetics data from clinical trials and pre-clinical research
Planet Microbe	https://www.planetmicrobe.org/	Oceanographic omics datasets linked to environmental metadata
Plant-ImputeDB	http://gong_lab.hzau.edu.cn/Plant_imputeDB	Reference panels and imputation methods for plant genomes
PROTAC-DB	http://cadd.zju.edu.cn/protacdb/	Proteolysis-targeting chimeras (PROTACs)
RASP	http://rasp.zhanglab.net/	RNA secondary structure probing data
RBP2GO	https://rbp2go.dkfz.de	RNA-binding proteins across species
RJunBase	www.RJunBase.org	RNA splice junctions
RMDisease	http://www.xjtlu.edu.cn/biologicalsciences/rmd	Genetic variants that affect RNA modifications vs disease
RMVar	http://rmvar.renlab.org	Genetic variants that affect RNA modifications vs disease
SC2disease	http://easybioai.com/sc2disease/	Single cell transcriptomics data and disease
SilencerDB	http://health.tsinghua.edu.cn/silencerdb or http://bioinfo.au.tsinghua.edu.cn/silencerdb/	Human silencers, validated or predicted
STAB	http://stab.comp-sysbio.org/	Spatio-Temporal Cell Atlas of the Human Brain
TBDB	https://tbdb.io	Structurally annotated T-box riboswitch: tRNA pairs
TCRdb	http://bioinfo.life.hust.edu.cn/TCRdb	T-cell receptor (TCR) sequences
ThermoMutDB	http://biosig.unimelb.edu.au/thermomutdb	Protein Mutation Thermodynamics Database
TISCH	http://tisch.comp-genomics.org/	Uniformly processed tumor (and microenvironment) scRNA-seq data
TransCirc	https://www.biosino.org/transcirc/	Protein coding potential of circRNAs
tRFtarget	http://trftarget.net	Targets of tRNA-derived fragments
tsRBase	http://tsrbase.org/	tRNA-derived small RNA expression and function
VARAdb	http://www.licpathway.net/VARAdb/	Variants and regulatory information

**Table 3.** Updated descriptions of databases most recently published elsewhere

Database name	URL	Short description
Bgee	https://bgee.org/	Curated wild-type animal gene expression data
CellMinerCDB	https://discover.nci.nih.gov/cellminercdb/	Cell line-based pharmacogenomics datasets
GeneLab	https://genelab.nasa.gov/	Omics data relating to space biology and ionising radiation
HMPDACC	https://portal.hmpdacc.org/	Human Microbiome Project Data Coordination Center
miRNASNP	http://bioinfo.life.hust.edu.cn/miRNASNP/	miRNA-related SNPs and mutations
MitImpact	https://mitimpact.css-mendel.it/	Precomputed pathogenicity predictions for human mitochondrial genome mutations
ModelSEED Biochemistry	https://modelseed.org/biochem	Biochemical reactions
PLncDB	http://plncdb.tobaccodb.org/	Plant long non-coding RNA
Project Score	https://score.depmap.sanger.ac.uk/	CRISPR–Cas9 screens to identify cancer dependencies
TREND-DB	http://shiny.imbei.uni-mainz.de:3838/trend-db/	Conditional alternative polyadenylation

As is customary, the Issue starts with reports from the major database providers at the U.S. National Center for Biotechnology Information (NCBI), the European Bioinformatics Institute (EBI) and the National Genomics Data Center (NGDC) in China (1–3). Thereafter, the usual categorisation applies: (i) nucleic acid sequence and structure, transcriptional regulation; (ii) protein sequence and structure; (iii) metabolic and signaling pathways, enzymes and networks; (iv) genomics of viruses, bacteria, protozoa and fungi; (v) genomics of human and model organisms plus comparative genomics; (vi) human genomic variation, diseases and drugs; (vii) plants and (viii) other topics, such as proteomics databases. Many resources are not easily pigeon-holed so browsing of the whole Issue is strongly encouraged.

The COVID-19 papers span a number of sections clearly indicating the multidisciplinary nature of the huge scientific response to the pandemic. Navigating the deluge of COVID-19 papers is a significant challenge in its own right and one addressed by the NCBI’s LitCovid database (4) which features manual curation supported by sophisticated machine-learning assistance. SARS-CoV-2 nucleic acid sequence data and associated curated metadata can be conveniently obtained from the ViruSurf database (5) which also covers other human pathogenic viruses. SARS-Cov-2 comparative genomics is covered by the GESS database (6) where temporal and geographical patterns of SNVs can be analysed. SARS-Cov-2 protein structures – alone and in complex with antibodies, receptors, and small molecules – are collected at the CoV3D database (7) and made available with a variety of bespoke analyses of sequential and conformational diversity. Obviously, drug and vaccine development are the primary drivers of SARS-Cov-2 protein structure determination. Supporting these efforts is the COVID19 Drugs Repository (8) which curates and integrates experimental findings from the literature with information from well-established small molecule databases to support drug repurposing. In a similar vein, DockCoV2 (9) contains *in silico* docking results for already approved drugs against putative drug targets in the SARS-Cov-2 proteome. Finally, recognising the therapeutic importance of an understanding of the host response, PAGER-CoV (10) covers pathways and gene lists relating to the viral infection and its consequences.

In the ‘Nucleic acid databases’ section, two significant returning databases focus on ncRNAs. The major news from the NONCODE database (11) is the inclusion of plant lncRNAs from 23 species, including their gene expression, function annotation and sequence conservation between species. NONCODE also reports new efforts to document associations between mammalian lncRNAs and cancers. MNDR (12) reports progress on a number of fronts—including the addition of circRNA-disease associations and ncRNA subcellular localization—resulting in a quadrupling of database entries. Rfam (13) reaches version 14 with new RNA families, some community-derived (including from masters students the paper reports) using a new Rfam Cloud platform, and including coronavirus and flavivirus entries. Integration with RNAcentral, also updating here (14), is evident in a new sequence search and work to make miRNA families more consistent with fellow member miRBase (15). RNAcentral continues to grow impressively, now encompassing 44 member ncRNA databases. Resources added since the last publication expand content in different directions, adding new classes of ncRNA such as snoRNAs, new links to diseases and new organism coverage.

Elsewhere, a number of databases focus on the structure, at small or large scale, of nucleic acids. The new TBDB (16) focuses on T-box riboswitch:tRNA pairs, where secondary structure modelling is required to predict functionality, while RASP (17) collects experimental data reporting on RNA secondary structure from a variety of sources including SARS-CoV-2. A major new arrival is the Nucleome Data Bank (18), which brings together a repository of experimental structural genomic data, computational tools for the modelling of structures, and visualisation of structures in the context of sequential data. They also introduce, by analogy with PDB files, an NDB file format. In the same area, 3DIV (19) reports an update focusing largely on 3D cancer genomes and commenting on the consequences for chromatin structure of the DNA structural variations associated with some cancers. Finally, DNAmoreDB (20) collects data on catalytic DNA molecules or DNAzymes, already demonstrated to catalyse 20 different reactions with that number sure to grow as research on their practical applications continues.

In the section on protein sequence and structure databases, users of protein family and domain databases are particularly well-served. COG (21) returns to report well over 200 new protein families and a similar number of families updated to reflect recent experimental characterisation. The current COG includes almost 5000 COGs and annotates genomes of over 1200 microbes. Similarly popular resources Pfam, SMART and Panther also report updates. Pfam (22) adds over 350 new families, including entries for previously unmatched SARS-CoV-2 proteins, and improves the consistency of its content with the specialist RepeatsDB resource, also updating here (23). A major strategic decision reintroduces the automatically generated Pfam-B supplement of non-curated sequence clusters calculated using more efficient computational methods. SMART (24), in contrast, does not aim for complete proteome coverage and the most recent targeted focus has been mobile genetic elements in bacteria and archaebacteria. The Panther update (25) reports an interesting new facility for assigning a function to a new sequence using tree grafting whereby Gene Ontology terms are attached to a protein according to the position at which it is best accommodated in the tree.

In the area of protein structure, the RCSB Protein Data Bank (26) reminds us that this venerable database turns 50 in 2021, having grown from just seven entries then (27), and distribution via magnetic tape, to ∼170 000 now. Its increasingly comprehensive analytical and visualisation features are exemplified using SARS-CoV-2 structures in the update paper. Another database with a long history, ProThermDB, covering thermodynamic parameters relating to protein stability, returns revitalised and near-doubled in size after 15 years (28). Two new databases ThermoMutDB (29) and FireProtDB (30) offer competition in the same area. For proteins without regular stable structures, the Issue offers a trio of databases. The main innovations at the returning database MobiDB (31) cover functional annotations relating to disordered regions; regions undergoing a disorder to order transition on binding, predicted linear interaction motifs, post-translational modifications and regions implicated in phase separation. PED (32), also publishing an update, covers experimentally characterized structural ensembles of disordered regions and proteins. These are joined by the newcomer MemMoRF (33) which covers features within disordered regions that can interact with biological membranes. Finally the Gene Ontology (34), a cornerstone resource across all sections, contributes an update describing how collaborations with expert groups and databases are being used to enrich, expand and improve the accuracy of the ontology.

In the metabolic and signalling section, the hugely popular STRING database of functional associations (35) offers an update that describes improvements to the functional enrichment tests available when users upload lists of proteins. STRING also now allows users to visualise and score all functional associations, as previously, or to limit visualisation and scoring to only physical interactions. The equally influential KEGG returns (36) with improved treatments of viruses and new visualisations, a more dynamic and interactive pathway map viewer and global map display with more flexible and informative colouring. A number of papers cover metabolic pathways. WikiPathways (37) reports increased numbers of pathways, metabolites and—importantly, given its modus operandi—contributors. Distinct portals with their own URLs support communities focused on topics as diverse as COVID-19, rare diseases and lipid metabolism. The popular ModelSEED Biochemistry database (38), publishing here for the first time, reports a doubling of content since its first iteration. Quantitative pathway modelling requires easy access to relevant data. The new database Datanator (39) steps in here to collect highly diverse data from a wide range of relevant resources and present it in a normalised and integrated fashion. Kinases are particularly well covered this year with KLIFS, the database for kinase–inhibitor interactions, reporting an update (40) that extends coverage to atypical kinases and provides an API for easy access. It is joined by two new resources, DKK (41) focussing on the ‘Dark Kinome’ and collecting information on substrates, inhibitor and tissue expression; and KinaseMD (42) which provides a detailed analysis of the impact of mutations on kinase structures and their consequences for drug binding. Finally, two new databases feature gene clusters. BiG-FAM (43), from the developers of the well known antiSMASH database (44), contains families of Biosynthetic Gene Clusters and enables a better understanding (and exploitation) of the full range of microbial natural product synthetic capacity. dbCAN-PUL (45) also builds on and complements an earlier database, in this case dbCAN-seq (46), and offers an online repository of experimentally characterised Polysaccharide Utilization Loci.

The microbial genomics section begins with a pair of databases devoted to anti-CRISPR (Acr) proteins. AcrHub (47) brings together over 300 experimentally characterised Acr proteins, 70 000 predicted Acr proteins and a variety of modules for characterising potential Acrs in user-uploaded sequences. AcrDB (48) detects and presents operons containing Acr-coding genes and nearby Aca regulatory proteins and uses machine learning to score them further. For comparative genomics, sister resources from the JGI, IMG/M and IMG/VR report updates. IMG/M (49) contains billions of genes from genomes and metagenomes and allows flexible mining and comparison between selected groups of sequences. IMG/VR (50) has near-tripled in size since its last update paper, its content dominated by 15 000 metagenomes that map across all continents and oceans. Also focused on viruses, VIPERdb (51), the database of viral capsids returns after a decade with new features including structure-based sequence alignments that allow identification of significantly conserved positions. Elsewhere metagenomics, and the human microbiome in particular, are a strong focus. Access to human metagenomes will be facilitated by the new curated metadata resource HumanMetagenomeDB (52) while another newcomer GIMICA (53) focuses on human genetic and immune factors that influence the human microbiota. The HMPDACC (54), published here for the first time, takes a multi-omics view of the human microbiome in health and disease, and encompasses 20 different data types. Finally, Planet Microbe (55) offers a home for oceanographic omics datasets, linking them to as much associated environmental context as possible.

In the next section, model organisms feature strongly. Flybase returns (56) to report many new features including Pathway Reports for major signalling pathways, better annotation of enzymes and flagging of proteins whose orthology to characterised human proteins renders them potentially relevant to disease. The Mouse Genome Database (57) also strongly emphasises the relevance to health and disease of comparisons with human proteins, and has a dedicated portal containing mouse research relevant to SARS-CoV-2. The ZFIN database (58) for zebrafish showcases newly designed pages driven by community feedback and describes the exchange of data with the Alliance of Genome Resources (59). Elsewhere two groups of organisms, arguably unfairly neglected hitherto, gain their own dedicated comparative genomics databases. MolluscDB (60) caters to members of the second largest animal phylum and includes not only 20 diverse and high-quality mollusc genomes, but also an array of functional genomic and even paleobiological data. Phycocosm (61) harbours genome (nuclear and plastid) and other omics data for the comparative study of algae. The UCSC Genome Browser (62) is a popular choice for interactive retrieval and display of genomes and associated tracks at its own site or embedded in other databases. Its response to the COVID-19 pandemic included rapidly making the SARS-CoV-2 sequence available with a variety of annotations, but also annotating human genome tracks with SNPs relevant to disease susceptibility and severity. Finally, the section also includes a trio of databases regarding ncRNA expression. The popular deepBase updates with a paper (63) describing a huge increase in expression datasets incorporated plus new and strong foci on ncRNA expression in cancer and exosomes. The new resource LncExpDB (64) intensively covers human lncRNA expression, including subcellular compartments and coexpression with potentially interacting mRNA molecules. Finally, LncSEA (65), also for human lncRNAs, supports research into lncRNA function by deriving reference sets of lncRNAs. Users can submit lncRNA lists for annotation and enrichment analyses.

The section on human genomic variation, diseases and drugs is again the largest in the Issue. Two new databases report on genetic variability in national populations. CSVS (66) reports on 2000 Spanish genomes and exomes and is notable for the crowd-sourcing from local projects behind its data, while IndiGenomes (67) covers 1000 Indian genomes from the country's notably diverse population.The GVM (68) collects genetic variation information from 41 species and contributes an update reporting a doubling in size and an analysis of thousands of SARS-CoV-2 variants. The new VARAdb database (69) comprehensively annotates human variants with a welcome emphasis on non-coding changes. Other databases link SNPs to specific molecular phenomena. Thus, RMDisease (70) and RMVar (71) each link genetic variants to RNA modifications and consider their potential impacts on disease. miRNASNP plays a similar role for SNPs that affect miRNAs or their targets and this update (72) reports a dramatic expansion and a strong focus on disease-related variants. Linking genotypes to phenotypes is the focus of two new databases PheLiGe (73) and Open Target Genetics (74). The latter's sister resource, the Open Targets Platform (75) for linking drug targets to diseases, contributes an update describing new scoring of drug targets from an increased range of contributing datasets. These new sources include Open Targets Genetics and Project Score, which includes results from CRISPR knockout screens of cancer models and which also features in this Issue (76). Other cancer databases include two new resources specifically focusing on cancer drivers, CNCdatabase (77) for non-coding cancer drivers and Oncovar (78) which complements experimental data with bioinformatically predicted drivers. canSAR, the multi-faceted oncology database reports new data, interface and query options (79). The paper guides the reader through the database, from the notably clear target synopsis pages to further information that would support a researcher in validating a target in a particular cancer. Among resources relevant to drug candidates, PubChem (80) is the major returning database, reporting over 100 new data sources enabling, for example, better links to literature, patents, material properties and toxicological data. Finally, given the explosion of interest in PROTACs for the targeted degradation of disease-related proteins, it's worth mentioning PROTAC-DB (81) which collects information on PROTACs’ structures, biological activities and drug-like properties.

Crops are, as usual, a major focus in the plant database section. SoyBase (82) returns with an update including new features for visualisation of gene expression and soya pan-genomes. The family is covered more broadly at LegumeIP (83) which now includes 17 legume genomes. A key focus is enabling translation of knowledge on agriculturally desirable traits from model genomes such as soybean to other species. A new database, Plant-ImputeDB (84), will also support crop breeding efforts by providing reference panels for 12 commercially important crops, thereby enabling better interpretation of genome variation. Two returning databases facilitate comparative plant genomics work. GreenPhylDB (85) adopts the concept of the pangenome in order to facilitate exploration of evolutionary scenarios within and between species. Gramene contributes a comprehensive update (86) covering expanded content across genomes, pathways and gene expression. It concludes with an interesting view of the number of collaborations of the enterprise with other databases, including the Alliance of Genome Resources (59). A multi-omics perspective of the model plant Arabidopsis is offered by the new resource AtMAD (87) which integrates genotypes, transcriptomes, methylomes and phenotypes for 620 accessions.

As ever, the final section contains a fascinating variety of databases that do not comfortably sit elsewhere. A single paper covers both ArrayExpress and Biostudies (88) and describes the migration of content from the former, that long outgrew its genesis (89) as a repository for microarray data, in favour of the more flexible and general possibilities of the latter. Glycans are covered by the returning GlyTouCan database (90) for glycan structures which brings improved submission and validation, and by the new GlycoPOST (91) which focuses on mass spectrometry of glycans and glycoproteins. Elsewhere NASA GeneLab (92) publishes in NAR for the first time and covers omics data obtained in space or simulated space conditions. Finally, MitoCarta, the popular focused resource for the mammalian mitochondrial proteome updates with curated sub-organelle localization and assignment of proteins to a set of 149 ‘MitoPathways’ (93).

## NAR ONLINE MOLECULAR BIOLOGY DATABASE COLLECTION

The ongoing COVID-19 crisis has shown the resilience of the scientific community: resources were rapidly reallocated as a response to the pandemic and enabled sequencing thousands of strains, tracking infection rates as the virus spread across the globe, structural biology etc. Making the most of the data generated through various streams naturally involves databases and their efforts are somehow reflected in our database with the seven new COVID-specific databases mentioned above but this is but a small fraction of the international effort fueling the number of publications on SARS-CoV-2 and coronavirus-related research (94).

The new normal reduced our travel schedules providing additional time for a major revamp of our NAR online Molecular Database Collection (accessible at https://www.oxfordjournals.org/nar/database/c/). In addition to the customary removal of obsolete databases, hundreds of entries were updated, corrected or expanded bringing the total collection to 1641 databases. We thank the authors for their support for our ongoing effort monitoring the listed resources. Among the hundreds of entries updated, many were due to direct communication with xose.m.fernandez@gmail.com providing a plain text file as defined in https://www.oxfordjournals.org/nar/database/summary/1.
